# Copy number variations and founder effect underlying complete *IL-10Rβ* deficiency in Portuguese kindreds

**DOI:** 10.1371/journal.pone.0205826

**Published:** 2018-10-26

**Authors:** Fabienne Charbit-Henrion, Bernadette Bègue, Anaïs Sierra, Sylvain Hanein, Marie-Claude Stolzenberg, Zhi Li, Sandra Pellegrini, Nicolas Garcelon, Marc Jeanpierre, Bénédicte Neven, Isabelle Loge, Capucine Picard, Jérémie Rosain, Jacinta Bustamante, Marc Le Lorc’h, Bénédicte Pigneur, Alicia Fernandes, GENIUS Group, Frédéric Rieux-Laucat, Jorge Amil Dias, Frank M. Ruemmele, Nadine Cerf-Bensussan

**Affiliations:** 1 INSERM, UMR1163 and Institut Imagine, Laboratory of Intestinal Immunity, Paris, France; 2 Paris Descartes University-Sorbonne Paris Cité, Paris, France; 3 Department of Paediatric Gastroenterology, Hepatology and Nutrition, Necker-Enfants Malades Hospital, Assistance Publique des Hôpitaux de Paris (AP-HP), Paris, France; 4 GENIUS group, Paris, France; 5 INSERM UMR1163 and Institut Imagine, Translational Genetic, Paris, France; 6 INSERM UMR1163 and Institut Imagine, Immunogenetics of Paediatric Autoimmunity, Paris, France; 7 Cytokine Signaling Unit, Institut Pasteur, INSERM 1221, Paris, France; 8 INSERM, Centre de Recherche des Cordeliers, UMR 1138 Equipe 22, Institut Imagine, Paris France; 9 Genetic Unit, Cochin Hospital, Assistance Publique des Hôpitaux de Paris (AP-HP), Paris, France; 10 Paediatric Haematology-Immunology and Rheumatology Unit, Necker-Enfants Malades Hospital, Assistance Publique des Hôpitaux de Paris (AP-HP), Paris, France; 11 Department of Paediatrics, Hôpital Charles-Nicolle, CHU Rouen, Rouen, France; 12 Study Centre for Primary Immunodeficiency, Necker-Enfants Malades Hospital, Assistance Publique des Hôpitaux de Paris (AP-HP), Paris, France; 13 Laboratory of Human Genetics of Infectious Diseases, Necker Branch, INSERM UMR 1163 and Institut Imagine, Necker Hospital for Sick Children, Paris, France; 14 St Giles Laboratory of Human Genetics of Infectious Diseases, Rockefeller Branch, Rockefeller University, New York, New York, United States of America; 15 Histology, Embryology and Cytogenetics Unit, Necker-Enfants Malades Hospital, Assistance Publique des Hôpitaux de Paris (AP-HP), Paris, France; 16 Centre of Biological Resources, Structure Fédérative de Recherche Necker, INSERM US24, CNRS UMS3633, Assistance Publique des Hôpitaux de Paris (AP-HP), and Institut Imagine, Paris, France; 17 Department of Paediatrics, Centro Hospitalar S. João, Porto, Portugal; Monash University, AUSTRALIA

## Abstract

Mutations in interleukin-10 receptor (IL-10R) genes are one cause of very early-onset inflammatory bowel disease with perianal lesions, which can be cured by hematopoietic stem cell transplantation. Using a functional test, which assesses responsiveness of peripheral monocytes to IL-10, we identified three unrelated Portuguese patients carrying two novel *IL-10RB* mutations. In the three patients, sequencing of genomic DNA identified the same large deletion of exon 3 which precluded protein expression. This mutation was homozygous in two patients born from consanguineous families and heterozygous in the third patient born from unrelated parents. Microsatellite analysis of the *IL10RB* genomic region revealed a common haplotype in the three Portuguese families pointing to a founder deletion inherited from a common ancestor 400 years ago. In the third patient, surface expression of IL-10R was normal but signaling in response to IL-10 was impaired. Complementary DNA sequencing and next-generation sequencing of *IL10RB* locus with custom-made probes revealed a ≈ 6 Kb duplication encompassing the exon 6 which leads to a frameshift mutation and a loss of the TYK2-interacting Box 2 motif. Altogether, we describe two novel copy number variations in *IL10RB*, one with founder effect and one preserving cell surface expression but abolishing signaling.

## Introduction

Mendelian bi-allelic loss-of-function mutations in the *IL10RA*, *IL10RB*, *IL10B* genes encoding the two chains of IL-10R or IL-10 respectively have been reported in approximately 100 children since 2009 [1–11]. Affected children typically develop severe colitis, perianal lesions and folliculitis within the first weeks or months of life. Refractoriness to immunosuppressive therapies is usual and can lead to colectomy. Early genetic diagnosis is necessary to perform hematopoietic stem cell transplantation (HSCT) which can cure the disease, avoid colectomy and also prevent the high risk of B cell lymphoma associated with this condition [12]. Interleukin 10 (IL-10) plays a central role in intestinal mucosal homeostasis via a direct regulatory effect on intestinal macrophages [13,14]. Using a functional test, which assesses the inhibitory effect of IL-10 on the production of inflammatory cytokines by peripheral monocytes [1], we identified three unrelated Portuguese patients carrying two novel *IL10RB* mutations. One mutation consisted in a large deletion of exon 3, which abolished protein expression. It was shared by the three patients and a founder effect could be demonstrated. The other mutation identified in one patient was a heterozygous ≈ 6 Kb duplication comprising the exon 6 that preserved IL-10Rβ surface expression but abolished TYK2 phosphorylation and downstream STAT3 and STAT1 signaling.

## Material and methods

### Patients

Patients were included in each center after obtaining informed written consent for functional and genetic studies from both parents. The study was approved by the local ethics committee (Comité de Protection des Personnes, Ile-de-France II, 2009–155).

### Functional studies

Peripheral blood mononuclear cells (PBMC) were isolated on Ficoll Hypaque, and Epstein-Barr virus (EBV)-cell lines were obtained according to standard procedures. Production of IL-8 by PBMC stimulated by lipopolysaccharide (LPS, Sigma, Saint-Quentin Fallavier, France) in the presence of IL-10 (RD systems, Lille, France) was analyzed by enzyme-linked immunosorbent assay (Human CXCL8/IL-8 Duo Set Kit, R&D systems) as described [1]. To analyze IL-10Rβ expression, 2.10^5^ PBMC were stained at 4°C for 30 minutes with PE-labelled anti-IL-10Rβ antibody, or IgG1 isotype (R&D systems), and CD45-APC, -CD3-PeCy7, -CD19-horizon V450 (BD Bioscience, Rungis, France) and -CD14-FITC (Milteny, Paris) antibodies. To analyze STAT3 phosphorylation, 1x10^6^ PBMC were stimulated with 25 ng/mL IL-6 or IL-10 (R&D systems) and surface-stained with the same antibody cocktail. After fixation and permeation, cells were labelled with anti-phosphorylated STAT3 antibody according to manufacturer’s instructions (BD Bioscience). Cells were analyzed on CANTO II (BD Biosciences) using the FlowJO software (TreeStar Inc, Ashland, Ore). To study TYK 2 phosphorylation, EBV cell lines were stimulated with 25 ng/mL IL-10 or IL-6 or with 100 pM IFNα for 15 minutes. Protein extracts (40 μg) were separated by SDS-PAGE gel, transferred to nitrocellulose membranes, and incubated with the indicated antibodies as in [15]. Anti-STAT1 antibody was from Millipore, anti-STAT3, anti-STAT1-P-Y701, anti-STAT3-P-Y705 and anti-TYK2-phospho-YY1054/55 were all from Cell Signaling Technology. To measure TYK2 content the in-house TYK2 monoclonal antibody (T10-2, Hybridolab, Institut Pasteur) was used.

### Genetic analyses

Genomic DNA and RNA were extracted from PBMC with QIAmp DNA Blood Mini Kit and RNA Extraction Mini kit respectively (QIAGEN, Courtaboeuf, France). cDNA was obtained with QuantiTect Reverse Transcription Kit (QIAGEN). Each exon of *IL10RA* (NM_001558) and *IL10RB* (NM_000628) was amplified from genomic DNA by polymerase chain reaction (PCR) as described [3]. For analysis of the exon 3 deletion of *IL10RB*, genomic DNA was amplified using two primers located in introns 2 and 3, respectively (forward: 5’-TAAACAGATGTGCCGTCCTC-3’; reverse: 5’-TGAGATAAGACTTCACTCTGGTCA-3’). For analyzing exon 6 duplication, *IL10RB* cDNA was amplified using two primers located in exons 4 and 7 (forward: 5’-CCCCCTGGAATGCAAGTAGA-3’; reverse: 5’-ACAAGGGCCAAGACCATCT-3’). PCR products were purified with the QIAquick kit (QIAGEN) and Sanger sequenced on a Genetic Analyzer 3500XL (Applied Biosystems, Foster City, USA). Segregation analysis of microsatellite markers that contain short tracks of dinucleotide repeats and flanking the *IL10RB* gene were performed using fluorescent primers. The polymorphic markers chosen from the Genethon Linkage Map [16] were: *D21S1252/AFM261ZG1*; *D21S1895/AFMB280XD9*; *D21S1254/AFM276ZA5*; *D21S1898/AFMB308XE5*; *D21S262/AFM198TC5*; *D21S1888/AFMA218WB1*; *D21S1908/AFMC016YG9*; *D21S263/AFM211ZG9*; *D21S1916/AFMA052TC5*; and *D21S269/AFM263XF5*. Primers sequences are available at the Working Draft of the Human Genome available at UCSC, Human Assembly (GRCh37/hg19). Amplified fragments were electrophoresed on a ABI PRISM 3500XL DNA Analyzer and analyzed using the GeneMapper Software 5 (Applied Biosystems, Foster City, CA). Heterozygote frequency and the size range of alleles for each microsatellite marker were available at the Centre d’Etude du Polymorphisme Humain (http://www.cephb.fr/cephdb/browser.php).

To further analyze the exon 6 duplication, we performed high-throughput sequencing of the entire *IL10RB* locus of the family 3 as described [17]. Briefly, genomic DNA (1 to 3 μg) from each individual was mechanically fragmented to a median size of 200 bp with a Covaris S2 Ultrasonicator. Double-stranded fragmented DNA (100 ng) was end-repaired, and adaptors containing a specific eight-base barcode were ligated to the repaired ends (one specific barcode per patient). DNA fragments were amplified by PCR to obtain final precapture barcoded libraries that were pooled at equimolar concentrations. Capture was performed with SureSelect reagents (Agilent), 750 ng of the pool of precapture libraries and home-made biotinylated probes obtained from bacterial artificial chromosome as a template. The single-stranded biotinylated DNA probes were designed to cover a ≈ 150 kb chromosomal region encompassing the *IL10RB* gene on chromosome 21 (chr21:34,638,665–34,669,539) according to the February 2009 human reference sequence (GRCh37hg19). During capture, barcoded library molecules complementary to the biotinylated beads were retained on streptavidin-coated magnetic beads and amplified by PCR to generate a final pool of postcapture libraries covering the targeted region. A pool of libraries (3 DNA samples from P3 and both parents and 1 from healthy control) covering a ≈150 kb region including the entire *IL10RB* gene was sequenced on an Illumina HiSeq2500 (paired-end sequencing 130x130 bases, high-throughput mode). Sequences were aligned with the GRCh37hg19 human genome with Burrows-Wheeler Aligner version 0.6.2.13 [18]. Copy number variations (CNVs) were analyzed as previously described [19]. For precise analyses of breakpoint, regions were analyzed for the presence of repetitive elements using the RepeatMasker track (http://www.repeatmasker.org/) in the UCSC genome browser (https://genome.ucsc.edu/) [20].

## Results

### Description of the patients

**Fig 1:**
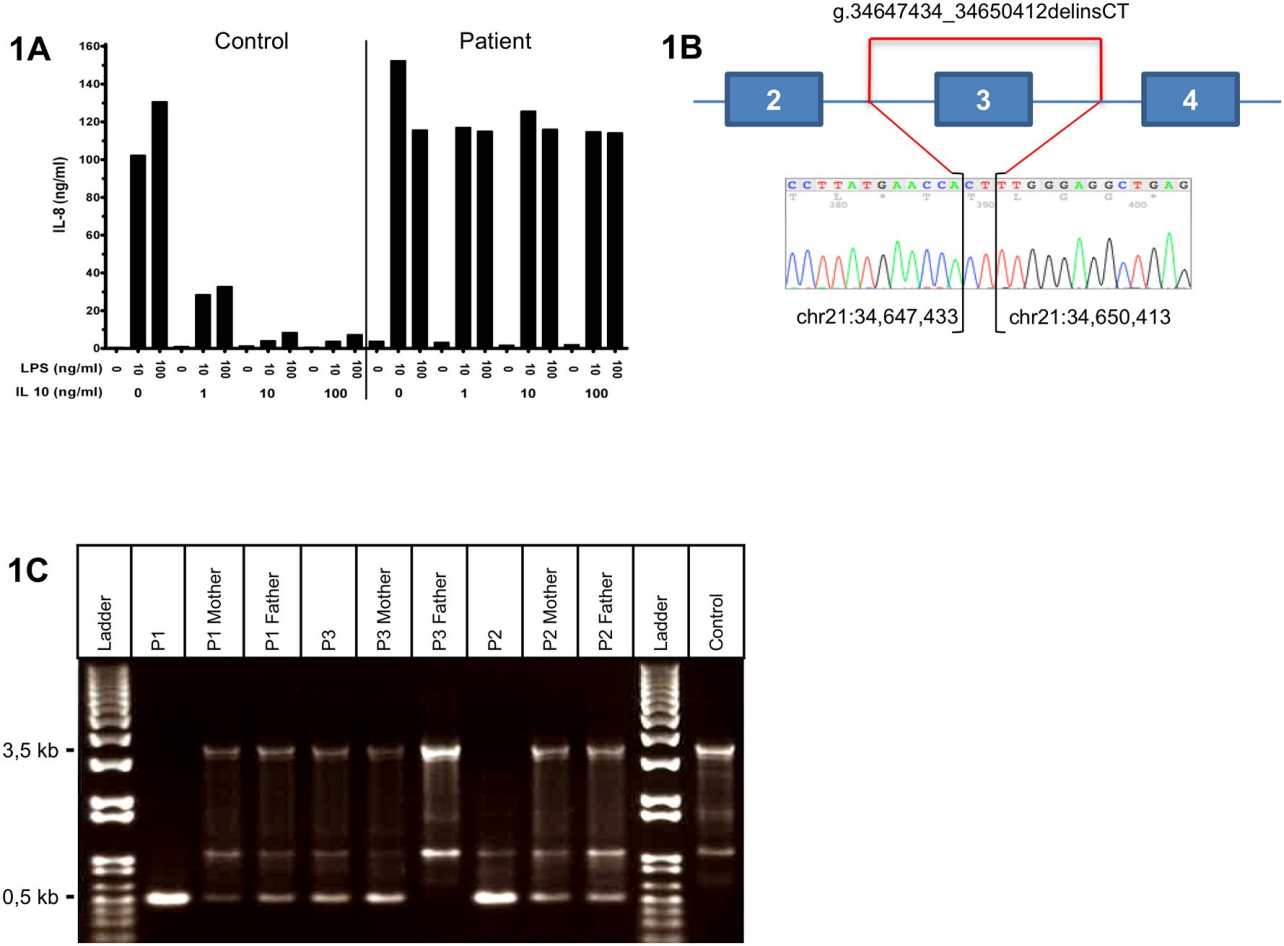
Patients from Portugal share a common deletion of exon 3 in *IL10RB*. 1A: Lack of responsiveness to IL-10 in PBMC stimulated with lipopolysaccharide (LPS) in Patient 3 compared to one control. IL-8 was quantified by ELISA in supernatants after a 24 hour-stimulation at the indicated concentrations of LPS and IL-10. Results were comparable in all three patients. 1B: Scheme showing the exon 3 deletion as defined by Sanger analysis. Genomic positions of breakpoints are shown by square brackets. 1C: Gel analysis of PCR products from genomic DNA with primers flanking the deletion showing the normal 3.5 kb band in control, parents and P3 and a short 0.5 kb mutated band in patients, parents of P1 and P2 and mother of P3.

Patients 1, 2 and 3 (P1, P2, P3) were born to three distinct Portuguese families, P1 and P2 to two consanguineous families while P3 was born from unrelated parents. The three patients presented with diarrhea, rectal bleeding, and perianal lesions since the age of 4 to 9 months. P1 also had severe chronic folliculitis. At diagnosis, endoscopy showed left colonic inflammation (P2) or pancolitis (P1 and P3). P1 was temporarily improved by ileostomy (S1 Table). This clinical phenotype was strongly suggestive of a defect in IL-10 signaling. The lack of inhibition by exogenous IL-10 of IL-8 production by PBMC stimulated by liposaccharide (LPS) further suggested mutations in the IL-10 receptor genes (Fig 1A).

### Identification of exon 3 deletion in *IL10RB* as a founder mutation

The receptor for IL-10 is a heterotetramer complex comprising two alpha chains encoded by *IL10RA* and two beta chains encoded by *IL10RB* [8]. In the three patients, genomic DNA sequencing did not reveal any mutation in *IL10RA* but pointed out an identical large deletion in *IL10RB* encompassing exon 3 in P1 and P2. Exon 3 deletion (delE3) was homozygous in P1 and P2 but heterozygous in P3. Sanger sequencing showed the loss of 2979 base pairs (bp) and the insertion of 2 bp (c.174-1467_331+1354delinsCT, g.34647434_34650412delinsCT, according to the HGVS v2.0 Nomenclature) (Fig 1B). Accordingly, the expected 3.5 kb band encompassing exon 3 and the flanking intronic regions were replaced in P1 and P2 by a 0.5 kb band carrying the deletion while P3 displayed both the 3.5 kb band and the abnormal 0.5 kb (Fig 1C). The parents of P1 and P2 and the mother of P3 displayed the two bands, indicating that they were heterozygous carriers of the deletion. Exon 3 skipping was predicted to result in a frameshift, which introduces an early-stop codon in exon 4 (p.Tyr59Hisfs*14).

In order to assess the role of genomic architecture in the mechanism of the large deletion of exon 3, we searched for known repetitive elements intersecting with the breakpoints. Breakpoints were localized respectively in a Tigger1 element (TcMar-Tigger family; DNA repeat element) in intron 2 (chr21:34,647,433) and in an *Alu*Jb/*Alu*Sx3 element (Alu family; short interspersed nuclear element / SINE) in intron 3 (chr21:34,650,413) (S1 Fig). Both are DNA repeat elements but they do not belong to the same family. Given the lack of homology and junctional microhomology and the 2 bp insertion, we suggest that the deletion of exon 3 may have been caused by non-homologous end-joining [21]. Of note, this deletion is described neither in DGV (http://dgv.tcag.ca/gb2/gbrowse/dgv2_hg19/?name=id:3146657;dbid=gene:database) [22], nor in gnomAD databases (www.gnomad.broadinstitute.org) [23].

The observation of the same large deletion in three unrelated Portuguese patients led us to suspect a founder effect. In keeping with this hypothesis, segregation analysis of microsatellite markers flanking *IL10RB* revealed a common haplotype from loci *D21S262* to *D21S1254* in all individuals carrying the delE3 (Table 1 and S2 Fig). Calculation of the putative age of the mutation was performed as described [24]. Assuming a linear mapping of 1.4 cM/Mb and given imprecision of the boundaries of presumably identical by descent sequences, time to a common ancestor was rounded to the nearest number of generations, giving a skewed distribution of 10–25 generations, with 15 as the mean. The size of the smallest region of homology was next used as a check on unrealistic calculations [24]. Assuming a star genealogy, in a naive bayesian framework, the sum of all branches given by algebraic expressions is 3/zx, with zx the size of the shortest region shared by all haplotypes. Supposing a shortest region of 3.5 cM, with these simplified assumptions, a comparable figure of 17 generations was obtained, overall suggesting that the mutation may have appeared approximately 400 years ago.

**Table 1:** Haplotypes analyses of the markers flanking the *IL10RB* gene.

															*IL10RB* chr21:34,638,665–34,669,539						
	*Centromere*	D21S269	0.98 Mb	D21S1916	3,24Mb	D21S263	0,03Mb	D21S 1908	0,6Mb	D21S 1888	0,96Mb	D21S 262	0,8Mb	D21S 1898 9		D21S 1254	1,28Mb	D21S 1895	1,47Mb	D21S 1252	*Telomere*
P1	Allele 1	251		244		175		216		271		148		233	**delE3**	264		266		249	
	Allele 2	251		242		175		216		273		148		233	**delE3**	264		270		231	
P2	Allele 1	241		242		175		216		277		148		233	**delE3**	264		266		247	
	Allele 2	251		242		175		216		277		148		233	**delE3**	264		266		249	
P3	Allele 1	243		244		201		218		271		148		233	**delE3**	264		274		239	
	Allele 2	243		248		199		218		271		148		215	**duplE6**	268		262		231	

Haplotypes reconstruction for informative microsatellite markers on chromosome 21q21.3-q22.12 of P1-3. Chromosomal start positions of each microsatellite markers are indicated in base pair (bp) according to the Human genome working draft sequence available from the University of California, Santa Cruz (UCSC, Human Assembly feb.2009 GRCh37/hg19). Physical distances in mega bases (Mb) between each marker are in red. D21S269 = AFM263XF5, 28.001.125bp; D21S1916 = AFMA052TC5, 28.981.056bp; D21S263 = AFM211ZG9, 32.221.915bp; D21S1908 = AFMC016YG9, 32.242.924bp; D21S1888 = AFMA218WB1, 32.854.278bp; D21S262 = AFM198TC5, 33.816.266bp; D21S1898 = AFMB308XE5, 34.609.209bp; D21S 1254 = AFM276ZA5, 35.075.320bp; D21S1895 = AFMB280XD9, 36.350.985bp; D21S1252 = AFM261ZG1, 37.826.859bp. Patient (P), Deletion of exon 3 of IL10RB (delE3), duplication of exon 6 (duplE6), and wild type allele (WT). Allele 1; maternal allele, Allele 2, paternal allele (see S2 Fig). Homozygous haplotypes are highlighted in grey and the common haplotype with the disease in the three families is framed.

### Identification of heterozygous exon 6 duplication in *IL10RB* impairing signaling but not protein expression

**Fig 2:**
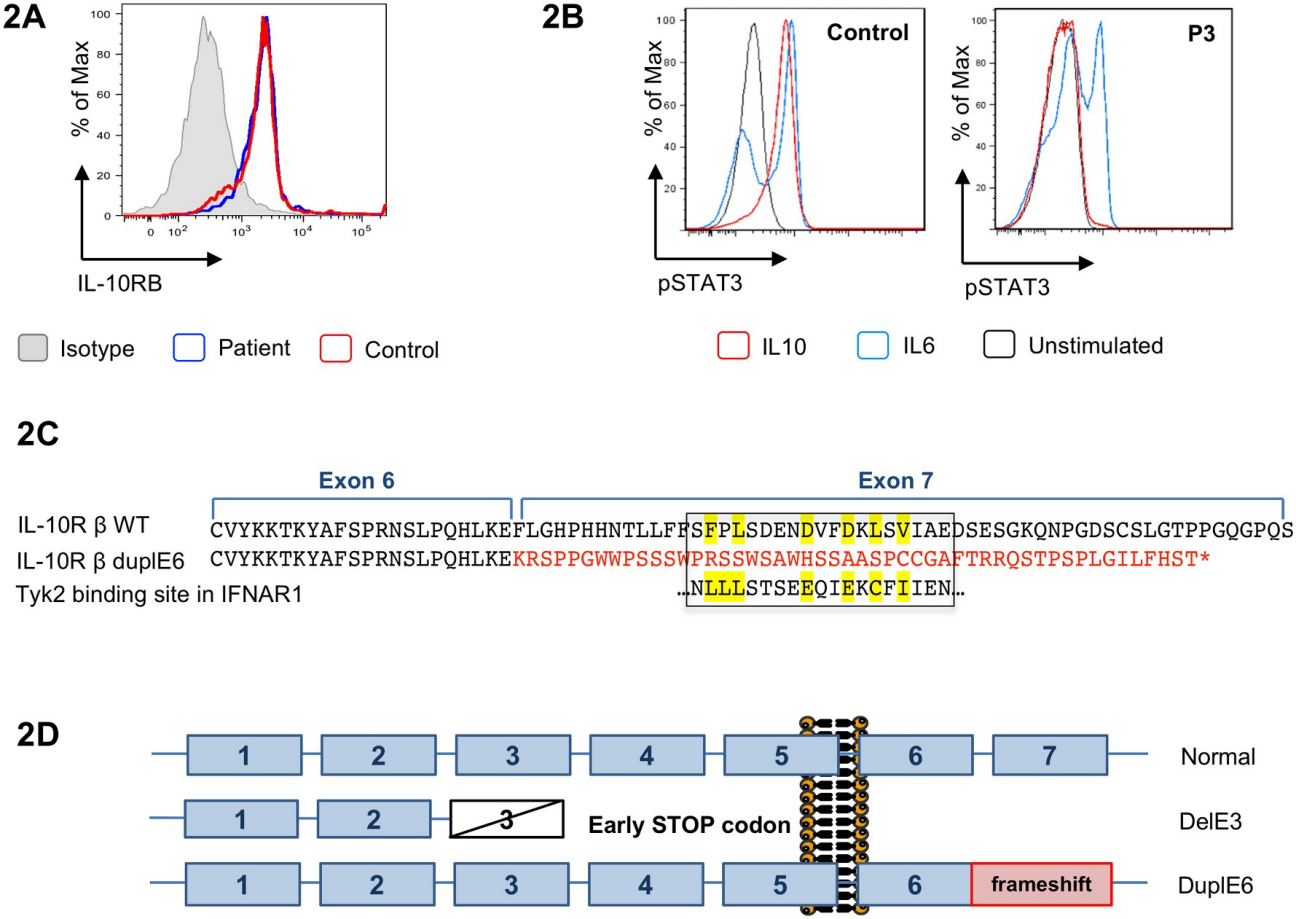
Duplication of *IL10RB* exon 6 results in loss of exon 7 encoded intracellular domain and prevents TYK2 activation. 2A: Flow cytometry analysis showing normal cell surface expression of IL-10Rβ in PBMC of P3 (gated on CD14^+^ cells). 2B: Flow cytometry analysis comparing STAT3 phosphorylation in response to IL-6 and IL-10 in PBMC of P3 and healthy control (gated on CD3^+^ cells). These data are representative of two independent experiments. 2C: Amino acid sequence of wild type IL-10Rβ and of the IL-10Rβ duplE6. In red, the aberrant 50 amino acid peptide resulting from the frameshift caused by the duplication of exon 6. Highlighted in yellow are residues conserved in IFNAR1, another TYK2-interacting cytokine receptor. 2D: Schematic representation of the two novel *IL10RB* mutations.

In P3, sequencing all exons of *IL10RB* on genomic DNA failed to reveal a second mutation. Moreover, flow cytometry revealed normal IL-10Rβ expression on PBMC (Fig 2A). These results contrasted with the lack of inhibition of IL-8 monocyte production by IL-10 (Fig 1A) and with the lack of STAT3 phosphorylation in P3’s PBMC stimulated with exogenous IL-10 while STAT3 phosphorylation was normal in response to IL-6 (Fig 2B). In order to identify the putative second mutation, sequence analysis of *IL10RB* was next performed on the cDNA from P3’s PBMC and both parents. Sanger sequencing of the PCR product amplified from P3’s cDNA with primers located in exons 4 and 7 revealed a heterozygous duplication of exon 6 (duplE6) (S3 Fig). This duplication was carried by the paternal allele (as shown by microsatellite analysis, data available on request). Unexpectedly it was detected in P3’s cDNA but not in her father’s cDNA. Long-range PCR on genomic DNA from P3 and her father using primers located in introns 5 and 7 failed to reveal the duplE6 (data not shown). We therefore performed next-generation sequencing of the entire *IL10RB* locus using custom made biotinylated probes on DNA from P3, both parents and one healthy wild-type control. Using both coverage and alignment NGS data, and in keeping with the result of the sequencing of P3’s cDNA, we identified a large insertion of ≈ 6 Kb encompassing the exon 6, which was present in P3 but also in her father (Part A in S5 Fig). In addition, our TNGS approach confirmed that P3 and her mother harbor the heterozygous delE3 (Part B in S5 Fig). DuplE6 could be localized approximately between breakpoints (chr21:34,657,300–34,657,450) in intron 5 and in intron 6 (chr21:34,663,650–34,663,870) (S4 Fig). Both breakpoints are localized in an *Alu*Sx1 repetitive element (*Alu* family; short interspersed nuclear element / SINE). Yet, their exact positions could not be determined precisely using either long-range PCR or PCR with primers framing the two breakpoints. Of note, duplE6 is described neither in DGV nor in gnomAD databases [22,23].

The consequence of duplE6 is a frameshift at the end of the first exon 6, which precludes translation of exon 7 and results in an aberrant sequence of 50 amino acids (Fig 2C). Accordingly, the mutated IL-10Rβ protein has intact extracellular and transmembrane domains, allowing expression at the cell surface (Fig 2A). However, the cytoplasmic domain is expected to contain the exon 6-encoded region (53 aa) followed by the aberrant C-terminal peptide (50 aa) (Fig 2C and 2D).

**Fig 3:**
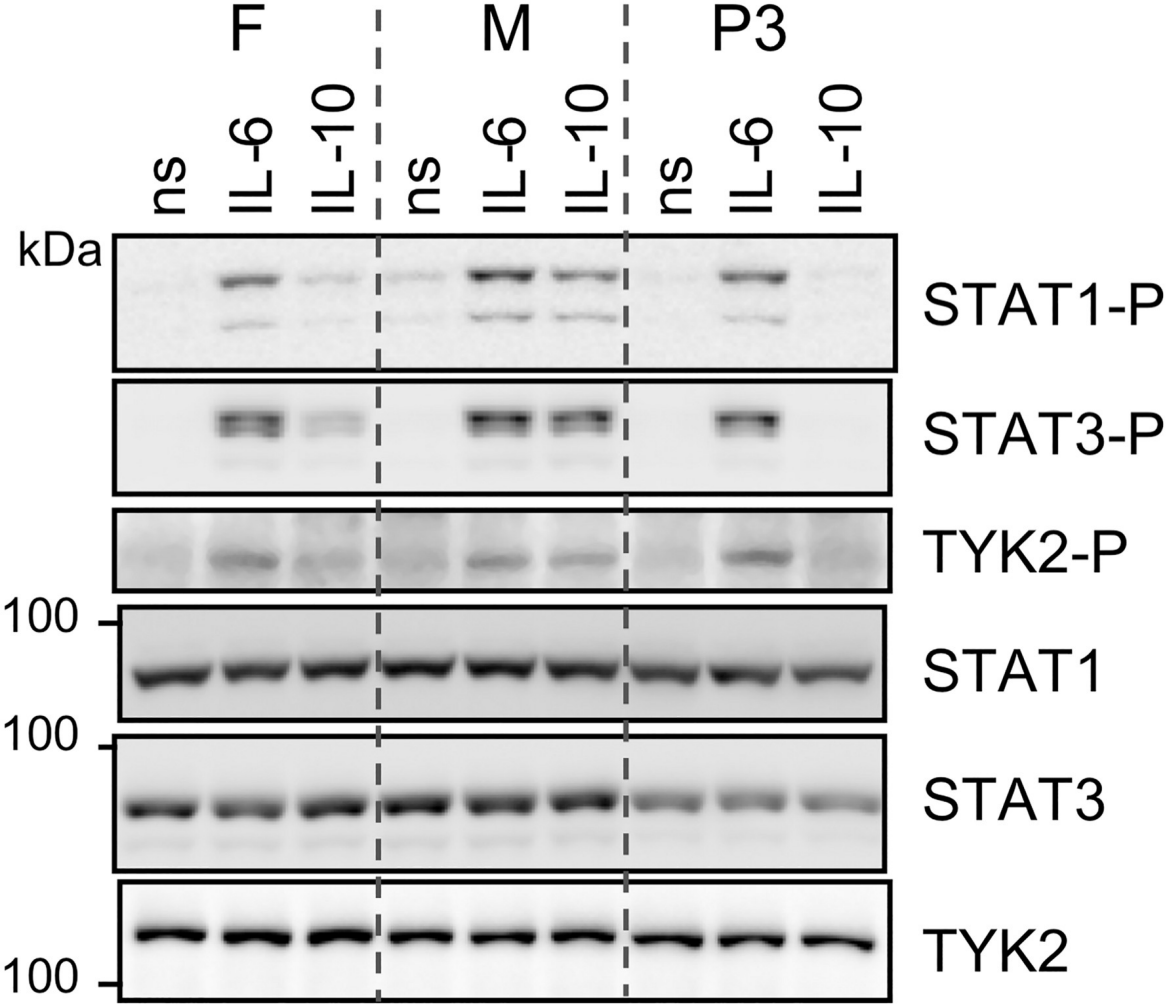
Lack of TYK2, STAT1 and STAT3 phosphorylation in P3’s EBV-B cells in response to IL-10. Western blot analysis of TYK2, STAT1 and STAT3 phosphorylation in EBV-B cells from P3 and her parents stimulated for 15 min with IL-6 or IL-10 (25 ng/ml). ns, non-stimulated. F, father. M, mother. Two gels were run in parallel with the same lysates. These data are representative of two independent experiments.

In the IL-10R complex, α and β chains are associated with two tyrosine kinases, JAK1 and TYK2 respectively. Binding of IL-10 to the receptor complex results in *trans*-phosphorylation of JAK1 and TYK2, recruitment and phosphorylation of the transcription factor STAT3 and to a lesser extent of STAT1 [25]. We analyzed signaling events in EBV-B cells derived from P3 and her parents. Cells were stimulated in parallel with IL-6 and IL-10 for 15 min and proteins were analyzed by western blot (Fig 3). Phosphorylation of TYK2, STAT1 and STAT3 in response to IL-6 was comparable in the three cell lines. On the other hand, phosphorylation of these proteins in response to IL-10 was undetectable in P3-derived cells (Fig 3). Of note, the latter cells expressed slightly lower levels of STAT3.

## Discussion

We report here three patients originating from Portugal with autosomal recessive complete IL-10Rβ deficiency. The combination of functional tests and genetic studies allowed us to describe two novel *IL10RB* copy number variations (CNV). One is a deletion and the first described founder *IL10RB* mutation; the second is a duplication preserving cell surface expression but abolishing downstream signaling.

To our knowledge, 23 patients have been reported in the literature with *IL10RB* mutations and CNV have been rarely identified. Only two patients carried CNV mutations. One was described by Pigneur et al (P5) and displayed a deletion of exon 2 [6]; the second was described by Kotlarz et al (P16) and carried a large deletion encompassing exons 4 to 7 (c.331+907_574del; [5]). No duplication has been reported so far. With the three patients reported here, deletion and duplication rates in *IL10RB* reach 19% (5/26) and 3.8% (1/26) respectively, with an overall rate of CNV of 23%. Thus, CNV rate in *IL10RB* (6/26, 23%) is higher than in the human genome (deletion: 7.5%; duplication: 3.9% and overall rate of CNV: 9.5%; [26]). This excess of CNV in the *IL10RB* locus may be due, at least partially, to its enrichment in *Alu* elements (24% of *Alu* elements *versus* 10% in the human genome [27]).

Interestingly, the same deletion of exon 3 was identified in three unrelated families, all originating from the same geographic origin. Even though *IL10RB* locus seems to be enriched in *Alu*-elements, the two delE3 breakpoints were non-homologous, reducing the probability that delE3 resulted from a recurrent rearrangement [21,28]. Furthermore, delE3 was absent from DGV and gnomAD databases. Altogether these data suggested that delE3 might be inherited from a common ancestor. In keeping with this hypothesis, all individuals carrying the delE3 shared a common haplotype from loci *D21S262* to *D21S1254*, a length compatible with an appearance approximately 400 years ago. To the best of our knowledge, delE3 is the first description of a mutation in *IL10RB or IL10RA* shared by a particular population.

Our observations in P3 illustrate difficulties in characterizing large CNV, and notably large duplications. Their identification requires combining several methods [21]. Thus, in P3, the large ≈ 6 Kb duplication encompassing exon 6 was not detected by Sanger sequencing of the genomic DNA but could only be identified by sequencing of the cDNA. Unexpectedly however, RT-PCR repeatedly failed to amplify the duplicated allele in P3’s father, suggesting a possible competition between the normal and duplicated allele. Overall, the duplication was best characterized using TNGS of the entire *IL10RB* locus captured with custom-made primers. TNGS, thanks to much higher coverage of each captured region of interest, emerges now as a powerful tool to identify CNV [27,29]. Of note, duplE6 could be easily detected using targeted sequencing of a panel covering the exons of *IL10RB* [29].

It is likely that P3 cells fail to respond to IL-10 due to the inability of the duplE6 mutant receptor chain to bind TYK2. In recent structural studies, Lupardus and coll. identified residues in other cytokine receptors that are critical for binding TYK2 and map in the so-called Box2 motif [30]. Inspection of the duplE6 cytoplasmic domain is consistent with the loss of Box 2, which in the WT IL-10Rβ is encoded by exon 7 (Fig 2C). Our data therefore suggest that the segment encoded by exon 7 is indispensable for signaling. Mutations preserving surface expression of IL-10R are uncommon and have been described only for *IL10RA* in two patients [31,32]. DuplE6 described here is the first example of a mutation preserving IL-10Rβ cell surface expression but impairing IL-10 signaling.

In conclusion, our results provide the first description of a founder *IL10RB* mutation. While its exact frequency in the Portuguese population remains to be assessed, it may increase the risk of IL-10Rβ deficiency either due to homozygous delE3 or to the combination of delE3 with a compound *IL10RB* mutation as observed in P3, who was born in a non-consanguineous family of Portuguese origin. Importantly, early identification of the molecular defect has been instrumental to indicate HSCT in all patients, which is currently the only definitive treatment. P3 unfortunately died of severe graft-versus host disease 12 months after HSCT while P1 and P2 are free of symptoms after 4.5 years and 3 years respectively.

## Supporting information

S1 Table Main clinical features of the three patients. (PDF)

S1 Fig Scheme depicting exon 3 deletion and breakpoints. (PDF)

S2 Fig Comparison of haplotype inheritance between the three Portuguese families. (PDF)

S3 Fig Sanger sequencing of cDNA showing exon 6 duplication in P3. (PDF)

S4 Fig Scheme depicting exon 6 duplication and breakpoints. (PDF)

S5 Fig TNGS analysis of the *IL10RB* locus in P3 and her parents. (PPTX)

## Abbreviations

bp: Base pairs
CNV: Copy number variations
delE3: Deletion of exon 3
dupE6: Duplication of exon 6
EBV: Epstein-Barr virus
ELISA: Enzyme-linked immunosorbent assay
EO-IBD: Early onset inflammatory bowel disease
FERM: Four-point-one, Ezrin, Radixin, Moesin
HSCT: Haematopoietic stem cell transplantation
IFN α: Interferon alpha
IFNAR1: Interferon alpha receptor 1
IL-10: Interleukin 10
IL-10R: Interleukin 10 receptor
JAK1: Janus kinase 1
LPS: Lipopolysaccharide
PBMC: Peripheral blood monocyte cells
SH2: Src Homology 2
STAT3: Signal transducer and activator of transcription 3
TNGS: Targeted next-generation sequencing
TYK2: Tyrosine kinase 2
