# Supplementary material for: Copy number variations and founder effect underlying complete IL-10Rβ deficiency in Portuguese kindreds
Source: PLoS One. 2018 Oct 26;13(10):e0205826. doi: 10.1371/journal.pone.0205826 (PMC6203366; doi:10.1371/journal.pone.0205826)
Supplement: S1 Table — (PDF) [file pone.0205826.s001.pdf]

S1 Table. Main clinical features of the three patients

P/DDB	Sex	Age at disease onset (months)	Perianal lesions	Endoscopy	Skin lesions	Surgery	Outcome
P1 2011	M	9	Yes	Pancolitis	Folliculitis	Ileostomy	HSCT in August 2013. Alive
P2 2013	M	5	Yes	Left colon and rectum inflammation	No	No	HSCT in November 2014. Alive
P3 2012	F	4	Yes	Pancolitis	No	No	HSCT in January 2015. Deceased in December 2015

P: patient; DDB: Date of birth; M: male; F: female; HSCT: hematopoietic stem cell transplantation
