# Supplementary material for: Copy number variations and founder effect underlying complete IL-10Rβ deficiency in Portuguese kindreds
Source: PLoS One. 2018 Oct 26;13(10):e0205826. doi: 10.1371/journal.pone.0205826 (PMC6203366; doi:10.1371/journal.pone.0205826)
Supplement: S2 Fig — (PDF) [file pone.0205826.s003.pdf]

S2 Fig

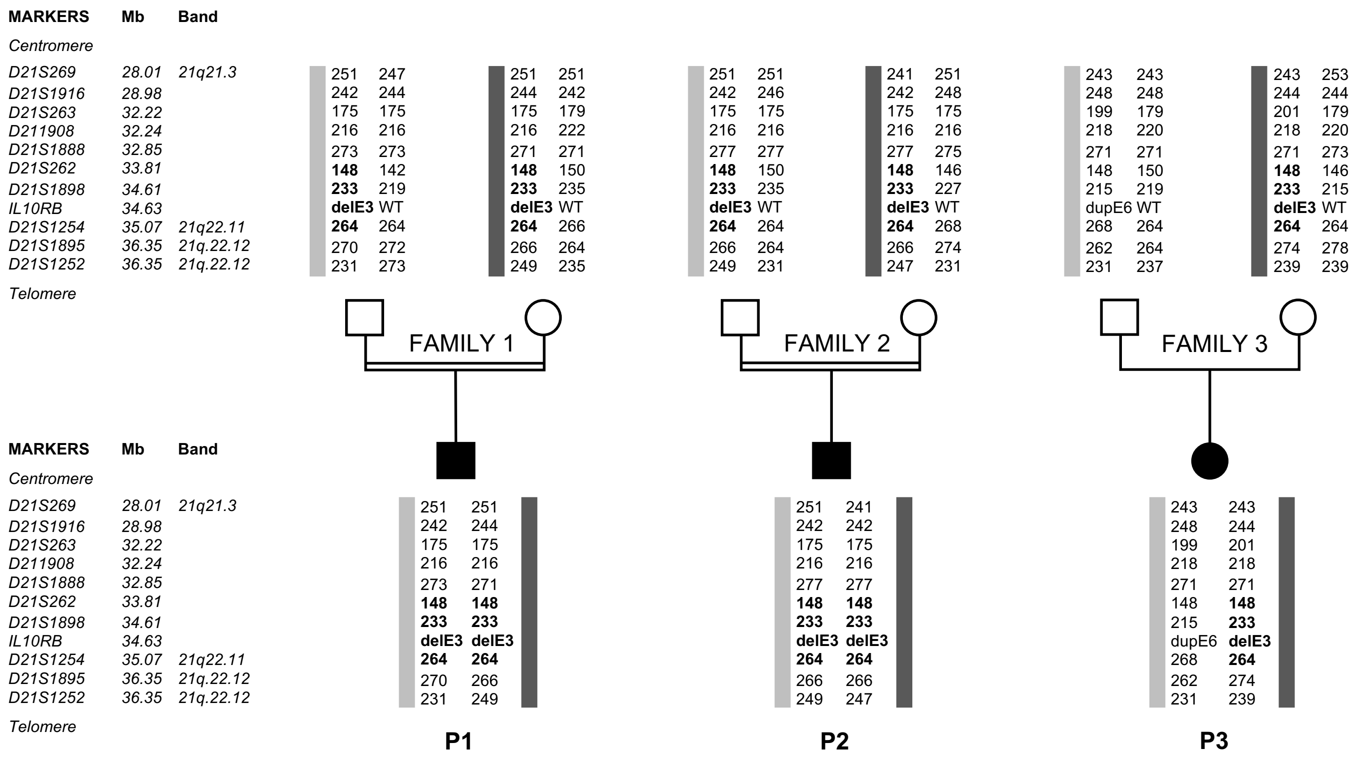

S2 Fig. Comparison of haplotype inheritance between the three Portuguese families. Haplotypes reconstruction for informative markers on chromosome 21q21.3-q22.13 of the three families. The ancestral haplotype shared by the three families is in bold.
