# Supplementary material for: Copy number variations and founder effect underlying complete IL-10Rβ deficiency in Portuguese kindreds
Source: PLoS One. 2018 Oct 26;13(10):e0205826. doi: 10.1371/journal.pone.0205826 (PMC6203366; doi:10.1371/journal.pone.0205826)
Supplement: S3 Fig — (PDF) [file pone.0205826.s004.pdf]

S3 Fig

A

CCCCCTGGAATGCAAGTAGAAGTACTTGCTGATTCTTTACATATGCGTTTCTTAGCCCCTAAAATTGAGAATGAATACGAAACTT
GGACTATGAAGAATGTGTATAACTCATGGACTTATAATGTGCAATACTGGAAAAACGGTACTGATGAAAAGTTTCAAATTACTCC
CCAGTATGACTTTGAGGTCCTCAGAAACCTGGAGCCATGGACAACTTATTGTGTTCAAGTTCGAGGGTTTCTTCCTGATCGGAA
CAAAGCTGGGGAATGGAGTGAGCCTGTCTGTGAGCAAACAACCCATGACG**AAACGGTCCCCTCCTGGATGGTGGCCGTCATC**
CTCATGGCCTCGGTCTTCATGGTCTGCCTGGCACTCCTCGGCTGCTTCGCCTTGCTGTGGTGCGTTTACAAGAAGACAAAGT
ACGCCTTCTCCCCTAGGAATTCTCTTCCACAGCACCTGAAAGAGAAACGGTCCCCTCCTGGATGGTGGCCGTCATCCTCATG
GCCTCGGTCTTCATGGTCTGCCTGGCACTCCTCGGCTGCTTCGCCTTGCTGTGGTGCGTTTACAAGAAGACAAAGTACGCCT
TCTCCCCTAGGAATTCTCTTCCACAGCACCTGAAAGAGTTTTTGGGCCATCCTCATCATAACACACTTCTGTTTTTCTCCTTTCC
ATTGTCGGATGAGAATGATGTTTTTGACAAGCTAAGTGTCATTGCAGAAGACTCTGAGAGCGGCAAGCAGAATCCTGGTGACAG
CTGCAGCCTCGGGACCCCGCCTGGGCAGGGGCCCCAAAGCTAGgctctgagaaggaaacacactcggctgggcacagtgacgtactccatctcacatct
gcctcagtgagggatcagggcagcaa**acaagggccaagaccatct**

B

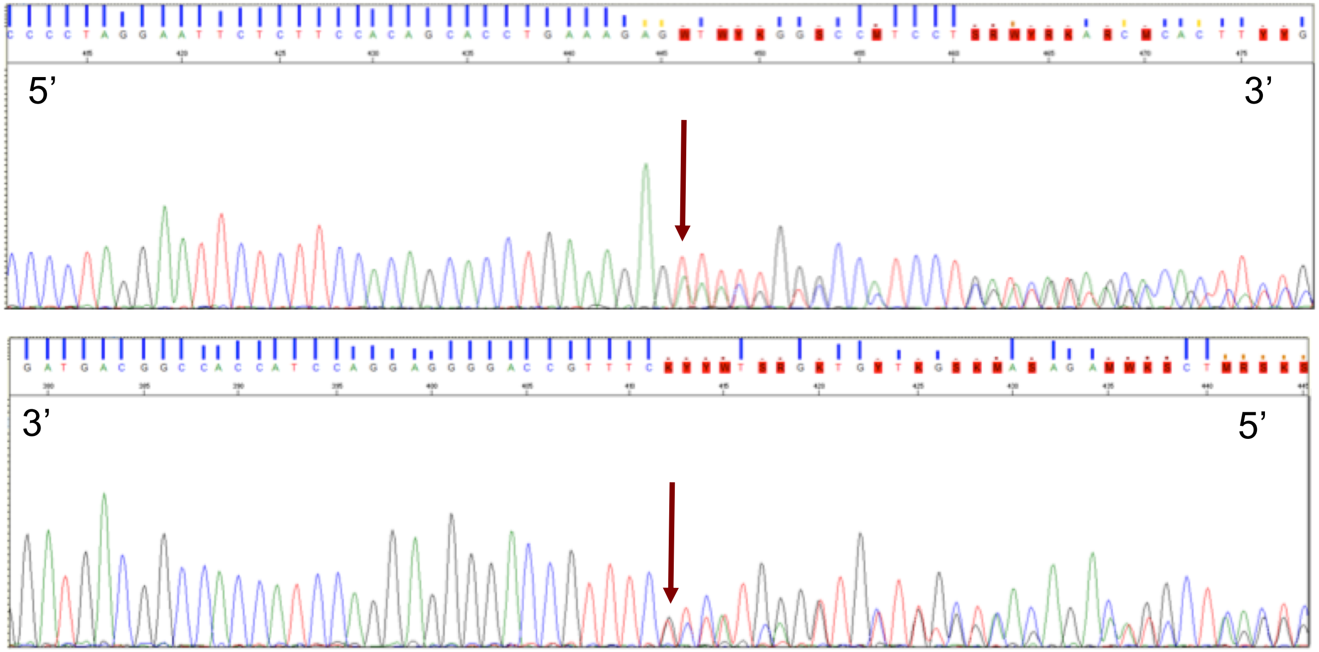

S3 Fig. Sanger sequencing of cDNA showing exon 6 duplication in P3. A- Sanger sequencing of PCR product amplified from P3’s cDNA with a forward primer in exon 4 (position 450-470 in cDNA) and a reverse primer in exon 7 (position 1327-1346) showing exon 6 duplication in the paternal allele. Primers are underlined. The first and second copy of exon 6 are shown in blue and red respectively. B. Electropherograms showing the overlap between the sequences of the second exon 6 on the paternal allele and of exon 7 on the maternel allele. The upper panel shows the 5’3’ sequences flanking the end of the first copy of exon 6 and the lower panel the 3’5’ sequences flanking the end of the second copy of exon 6. Red arrows indicate the nucleotide where the sequences inherited from the mother and father diverge.
