# Supplementary material for: Copy number variations and founder effect underlying complete IL-10Rβ deficiency in Portuguese kindreds
Source: PLoS One. 2018 Oct 26;13(10):e0205826. doi: 10.1371/journal.pone.0205826 (PMC6203366; doi:10.1371/journal.pone.0205826)
Supplement: S4 Fig — (PDF) [file pone.0205826.s005.pdf]

S4 Fig

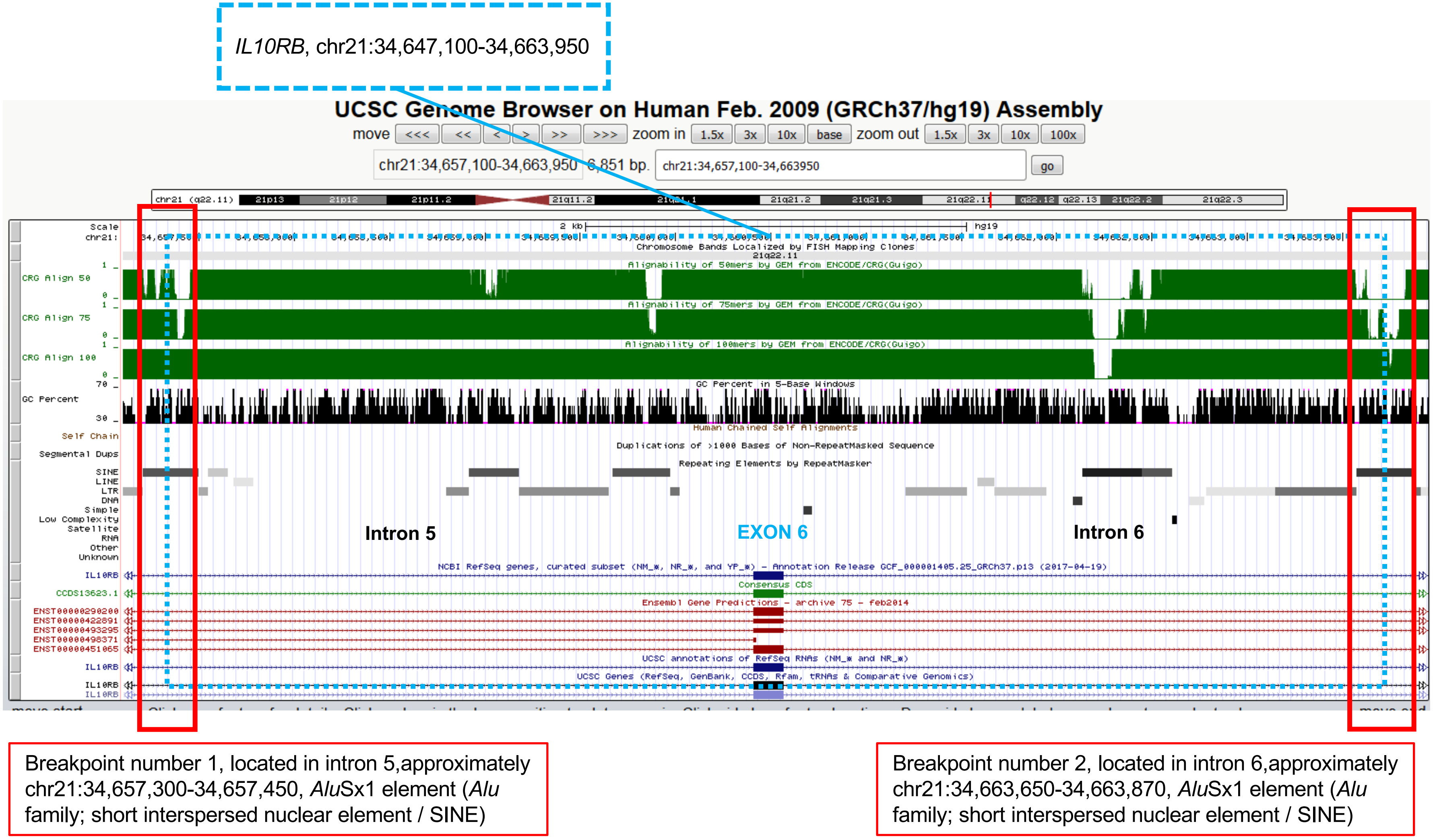

S4 Fig. Scheme depicting exon 6 duplication and breakpoints. Schematic representation was made with UCSC genome browser (https://genome.ucsc.edu/) using the RepeatMasker track (http://www.repeatmasker.org/).
