# Supplementary material for: Copy number variations and founder effect underlying complete IL-10Rβ deficiency in Portuguese kindreds
Source: PLoS One. 2018 Oct 26;13(10):e0205826. doi: 10.1371/journal.pone.0205826 (PMC6203366; doi:10.1371/journal.pone.0205826)
Supplement: S5 Fig — (PPTX) [file pone.0205826.s006.pptx]

## Slide 1
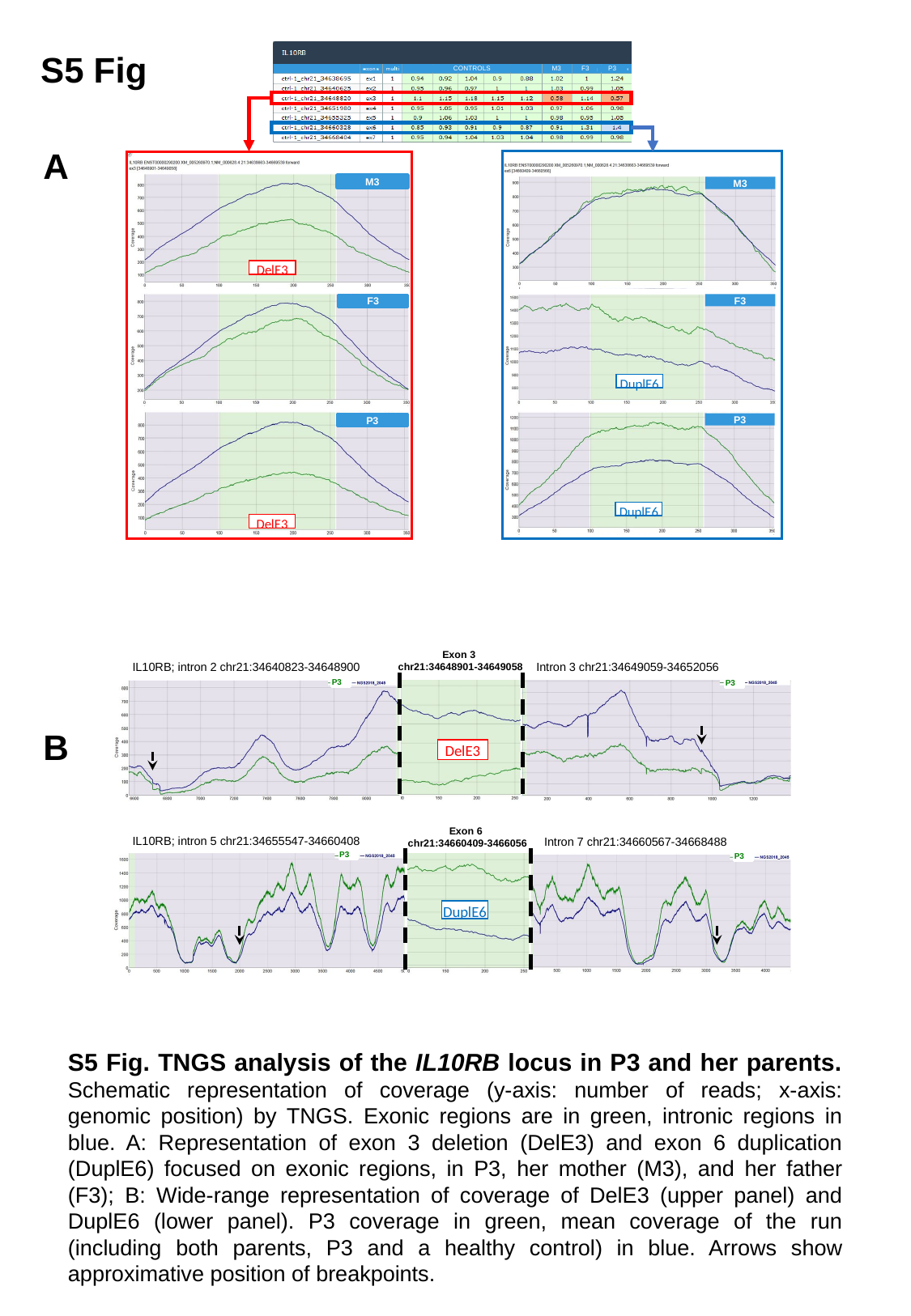

CONTROLS
M3
F3
P3
S5 Fig
A
M3
M3
DelE3
F3
F3
DuplE6
P3
P3
DuplE6
DelE3
Exon 3
chr21:34648901-34649058
Intron 3 chr21:34649059-34652056
IL10RB; intron 2 chr21:34640823-34648900
P3
P3
B
DelE3
Exon 6
chr21:34660409-3466056
IL10RB; intron 5 chr21:34655547-34660408
Intron 7 chr21:34660567-34668488
P3
P3
DuplE6
S5 Fig. TNGS analysis of the IL10RB locus in P3 and her parents. Schematic representation of coverage (y-axis: number of reads; x-axis: genomic position) by TNGS. Exonic regions are in green, intronic regions in blue. A: Representation of exon 3 deletion (DelE3) and exon 6 duplication (DuplE6) focused on exonic regions, in P3, her mother (M3), and her father (F3); B: Wide-range representation of coverage of DelE3 (upper panel) and DuplE6 (lower panel). P3 coverage in green, mean coverage of the run (including both parents, P3 and a healthy control) in blue. Arrows show approximative position of breakpoints.
